# Effect of sand-based training on sprint performance: a systematic review and meta-analysis

**DOI:** 10.3389/fphys.2026.1665495

**Published:** 2026-02-16

**Authors:** Kun Meng, Yunji Chen, Xu Xiang, Guole Jiang, Yang Liu, Qing Yi

**Affiliations:** 1 School of Physical Education, Hunan First Normal University, Changsha, Hunan, China; 2 Basic Education College, National University of Defense Technology, Changsha, Hunan, China; 3 Division of Sports Science and Physical Education, Tsinghua University, Beijing, China

**Keywords:** meta-analysis, plyometric training, sand-based training, sprint performance, training surfaces

## Abstract

**Background:**

Sand-based training (SBT) is widely hypothesized to enhance sprint performance; however, its overall efficacy remains unclear due to inconsistencies in methodologies and findings across studies. This systematic review and meta-analysis aims to evaluate the magnitude of SBT’s impact on sprint performance in competitive athletes.

**Methods:**

Following PRISMA guidelines, five databases (PubMed/MEDLINE, Web of Science, Cochrane Library, SPORTDiscus, and Scopus) were systematically searched from inception to May 2025. We included randomized controlled trials (RCTs) examining competitive athletes undergoing SBT interventions (≥4 weeks) compared to non-sand or no-intervention controls. The primary outcome was linear sprint performance. Meta-analyses were conducted using RevMan 5.3 and Stata 16.0; standardized mean differences (SMD) with 95% confidence intervals (CI) were calculated using random- or fixed-effects models.

**Results:**

Nineteen studies (N = 433 athletes) met the inclusion criteria. SBT significantly enhanced sprint performance in within-group (SMD = −0.92 [95% CI: −1.10, −0.74]; p < 0.001) and between-group comparisons (SMD = −0.64 [-0.87, −0.42]; p < 0.001). Subgroup analyses indicated that SBT demonstrated significantly greater improvements compared to alternative training modalities (SMD = −1.13, p = 0.001). In-season training and higher-frequency training (≥3 sessions/week) were associated with larger performance gains (SMD = −0.87 and −1.12, respectively).

**Conclusion:**

Current evidence suggests that SBT is a promising strategy for improving sprint performance, with maximal benefits observed when implementing high-frequency protocols during the competitive season. Future research should prioritize standardized training methodologies, long-term adaptive responses, and applicability across diverse athletic populations.

**Systematic Review Registration:**

https://www.crd.york.ac.uk/prospero/display_record.php?ID=CRD42025637332, identifier: CRD42025637332.

## 1 Introduction

Successful athletic performance is determined by the integration of multiple physical capacities, including change-of-direction ability, sprint performance, aerobic capacity, and strength/power levels (Carlsson et al., 2016; Freitas et al., 2023; Loturco et al., 2019; Suchomel et al., 2016). Among these, sprint performance is widely regarded as one of the most critical physical attributes (Faude et al., 2012; Menaspà et al., 2013; Scanlan et al., 2012). For instance, in elite soccer, nearly 50% of goal-scoring opportunities are preceded by direct linear sprints (Haugen et al., 2014). Similarly, short-distance sprinting is an essential for breaching defensive lines, securing advantageous positions, or executing crucial defensive actions across various sports (Faude et al., 2012; Menaspà et al., 2013; Scanlan et al., 2012). Notably, as competitive standards rise, professional athletes demonstrate progressive improvements in sprint capabilities (Haugen et al., 2013), alongside a significant increase in the frequency of high-intensity efforts during competition (Barnes et al., 2014). Consequently, the efficient enhancement of sprint capacity remains a priority in modern athletic conditioning.

In light of the increasing emphasis on sprint performance, practitioners and researchers continuously explore novel training methodologies to enhance sprinting velocity. Various approaches, such as plyometric training, resisted sprinting, and acceleration training, have proven effective in enhancing sprint speed (Freitas et al., 2019; Loturco et al., 2017; Loturco et al., 2015; Singh et al., 2022). These interventions are predominantly performed on firm surfaces (e.g., concrete, wood, or synthetic flooring) (Loturco et al., 2015; de Villarreal et al., 2009; Singh et al., 2022). The underlying rationale is that firm surfaces provide a greater stimulus to the stretch-shortening cycle (SSC), facilitating efficient storage and subsequent utilization of elastic energy during the eccentric muscle contraction phase. This enhanced elastic recoil ultimately augments power output during movement (Flanagan et al., 2007). However, such high-impact training may also increase joint loading and is associated with a higher risk of sports-related injuries (Binnie et al., 2014a; Pinnington et al., 2005).

In recent years, sand-based training (SBT) has gained attention as a potentially effective strategy for improving sprint performance. For instance, studies indicate that sport-specific sprint training (e.g., repeated sprints, change-of-direction runs, acceleration drills) conducted on sand effectively enhances short-distance sprint speed in soccer players (Impellizzeri et al., 2008). Plyometric training on sand has been shown to improve acceleration and maximal velocity in rugby athletes (Ramirez-Campillo et al., 2020), while periodized sand training in beach volleyball players is strongly correlated with superior on-court speed performance (Bozzini et al., 2021). From a neuromuscular perspective, training on sand significantly elevates activation levels of agonist muscles during targeted movements (Miyama and Nosaka, 2004; Pinnington et al., 2005), enhancing neuromuscular coordination and muscular control (Gaudino et al., 2013). The unique properties of sand attenuate ground impact forces, effectively reducing stress on joints and muscles compared to firm surfaces (Impellizzeri et al., 2008; Miyama and Nosaka, 2004). Practically, the widespread availability of sand resources (e.g., natural beaches or artificial courts) renders SBT a practical and economical training option.

Current research findings regarding the impact of SBT on sprint performance remain inconsistent. For example, Mehrez et al. (2022) reported that a 7 week in-season SBT program (3 sessions/week) significantly improved 5 m, 10 m, and 20 m sprint times in elite handball players. Conversely, Impellizzeri et al. (2008) found no significant improvement in 10 m or 20 m sprint times following a 4 week pre-season SBT program (3 sessions/week) in top-level soccer players. These discrepancies may stem from variations in training program parameters (e.g., frequency, intensity, duration, periodization) or differences in athletes’ training backgrounds, adaptability, and competitive levels.

Although numerous primary studies have investigated SBT’s effects on sprint performance (Guo et al., 2024; Sanchez-Sanchez et al., 2020; Slimani et al., 2016), significant heterogeneity exists in the current evidence base. This heterogeneity primarily arises from variations in training protocol parameters (e.g., frequency, duration, intensity, periodization phase) and participant characteristics (e.g., sport, competitive level), complicating the formulation of universal conclusions. While existing systematic reviews have attempted to synthesize evidence—such as Sanchez-Sanchez et al. (2020) comparing immediate/short-term performance differences across surfaces, and Slimani et al. (2016) evaluating plyometric training effects (mentioning non-firm surfaces including sand)—these reviews have not systematically addressed critical questions: (1) What is the true effect of SBT as a ≥4 week intervention on sprint performance? (2) How does the long-term effectiveness of SBT in improving sprint performance compare to other common terrestrial training surfaces (e.g., grass, hard ground)? (3) How do key moderating factors (e.g., training frequency, training cycle) influence SBT outcomes? Therefore, this study aims to address these evidence gaps through a systematic review and meta-analysis, with the following objectives: (1) Quantitatively synthesize existing high-quality evidence to determine the true effect size of SBT on sprint performance; (2) Compare the effectiveness of SBT against other terrestrial training modalities; (3) Identify the influence of key moderators (training frequency, periodization phase, athlete level) on SBT outcomes via subgroup analysis, thereby providing evidence-based guidance for optimizing SBT protocols in practice.

## 2 Materials and methods

This systematic review and meta-analysis was conducted in accordance with the Preferred Reporting Items for Systematic Reviews and Meta-Analyses (PRISMA) 2020 statement (Page et al., 2021) and the methodological guidelines outlined in the Cochrane Handbook for Systematic Reviews of Interventions (Higgins and Green, 2008). The study protocol was prospectively registered with the International Prospective Register of Systematic Reviews (PROSPERO: CRD42025637332).

### 2.1 Search strategy

A comprehensive systematic search was conducted across five electronic databases: PubMed/MEDLINE, Web of Science, Cochrane Library, SPORTDiscus, and Scopus. The search timeframe spanned from each database’s inception to 7 April 2025, with an updated search performed on 21 May 2025. Adhering to the PICOS framework (Population, Intervention, Comparison, Outcome, Study) (Page et al., 2021), we identified keywords through a preliminary scoping search to delineate terms associated with the target population, intervention, and performance outcomes. Key terms included “sand training”, “sand-based exercise”, “sand surface”, “beach training”, “athletes”, “players”, “sprint*”, “running speed”, “acceleration”, and “short-distance running”. These terms were combined using the Boolean operators AND and OR. Detailed search strategies for each database are provided in S2 File. Additionally, we manually screened Google Scholar and the reference lists of all eligible articles and reviews to identify further relevant studies.

### 2.2 Eligibility criteria

Inclusion criteria were defined based on the PICOS (Population, Intervention, Comparison, Outcome, Study design) framework, as detailed in Table 1. Studies were excluded if they: (1) involved non-athletes; (2) were published in languages other than English; (3) lacked sufficient data to calculate effect sizes (ES) and 95% confidence intervals (CI); (4) were reviews, conference proceedings, or non-original research; or (5) had unavailable full texts or were duplicate publications.

**TABLE 1:** Eligibility criteria for inclusion of studies.

Category	Inclusion criteria
Participants	The participants were athletes, with no restrictions on age, gender and level
Intervention	The intervention group must engage in a training program conducted exclusively on sand. The intervention should have a minimum duration of 4 weeks, with consistent training protocols (e.g., frequency, intensity, volume) across the duration
Comparator	The comparator group must follow exercise training principles, with training conducted on surfaces other than sand (e.g., hard ground, grass, synthetic surfaces), or be a no-intervention control group (i.e., participants who do not undergo any physical training during the study period). The intervention duration for the comparator group must be ≥ 4 weeks. If it is a non-sand training comparator group, training intensity, frequency, and duration should be comparable to the intervention group. The no-intervention group should experience no training or any form of exercise intervention during the same period
Outcome	The primary outcome must be linear sprint performance, measured using one or more of the following: Sprinting speed (e.g., m/s), sprinting time (e.g., seconds for a defined distance, such as 5 m, 10 m, 20 m, 30 m, 40 m, 50 m and 60 m, etc.)
Study design	Only randomized controlled trials (RCTs) are eligible. Studies must include at least two groups one experimental (sand training) and one comparator group (non-sand training or no-intervention group) and must include baseline and post-intervention measurements of sprint performance

### 2.3 Selection process

The retrieved studies were aggregated and stored in EndNote 20 software. After duplicate studies were removed, two reviewers (KM and YC) read the titles and abstracts of the remaining studies separately according to the inclusion and exclusion criteria, excluded the literature that did not meet the inclusion criteria, and then read the full texts of the literature that might meet the inclusion criteria to further judge whether they were included. Finally, the reviewers conducted face-to-face communication and proofreading of the final included studies. If the two reviewers disagree with the results of a study or eventual inclusion, it would be solved through discussion or consultation with a third reviewer (QY).

### 2.4 Data collection process

The data extraction process was conducted by two authors according to the Cochrane Collaboration Handbook (Higgins and Green, 2008). The data were extracted as follows.

- The first author, publication time of the literature, and publishing country/location.
- Average age, sex, and competitive level and sample size of the research subjects.
- Frequency, time, type of exercise, period of interventions, and sand depth.
- Outcomes: sprinting speed (e.g., m/s), sprinting time (e.g., seconds over defined distances), and testing protocols.
- Key information for risk assessment of bias

For each included study, the mean and the SD of the pre-tests, post-tests and follow-up tests were extracted. If any relevant data were missing, the corresponding author or authors were contacted via email.

### 2.5 Risk of bias assessment

Two independent investigators assessed the risk of bias for each included study using the Cochrane Collaboration’s tool (Higgins et al., 2011), strictly adhering to the seven-domain framework outlined in the Cochrane Handbook for Systematic Reviews of Interventions (Version 5.1.0). Disagreements between reviewers were resolved through discussion or consultation with a third reviewer.

The overall risk of bias for each study was categorized as Low, Moderate, or High. This classification was based on a comprehensive evaluation of all seven domains, strictly applying the criteria proposed by Cipriani et al. (2018): Low risk: No domain was judged as “High risk”, and ≤3 domains were rated “Unclear risk”. Moderate risk: Either (1) ≥ 1 domain was judged “High risk”, or (2) no domain was “High risk”, but ≥4 domains were rated “Unclear risk”. High risk: All other cases (e.g., ≥2 domains judged “High risk”).

### 2.6 Statistical analysis

RevMan 5.3 software (Cochrane Collaboration, Oxford, United Kingdom) and Stata version 16.0 (Stata Statistical Software, release 16; Stata Corp., College Station, TX, United States) were used for data analysis. Continuous data were analyzed by combining the mean difference (MD) of each study when the outcome was reported using the same measurement units; or the standardized mean difference (SMD) when the outcome was reported using different measurement units. Specifically, the MD was calculated as the mean difference of the outcomes in the intervention group before and after the intervention minus the mean difference of the outcomes in the control group before and after the intervention (Cohen, 1988). The SMD was then calculated as the MD divided by the pooled intervention-specific standard deviation. For studies reporting the MD and standard error (SE), we convert the rest studies with mean ± SD into MD and SE for the next analysis. The magnitude of SMD was classified according to the following scale: 0∼0.19 represents negligible effect, 0.2∼0.49 represents a small effect, 0.5∼0.79 represents moderate effect, and ≥0.8 represents large effect (Cohen, 1988). Value of p < 0.05 was considered statistically significant.

The I^2^ statistic was used to assess the extent of heterogeneity: negligible or small heterogeneity (0∼40%), moderate heterogeneity (30∼60%), substantial heterogeneity (50∼90%), and considerable heterogeneity (>75%). If heterogeneity was not significant (I^2^ < 50%), the fixed-effects model was adopted. If heterogeneity was significant (I^2^ ≥ 50%), a random-effects model was used (Bown and Sutton, 2010).

The meta-regression analysis was used to determine if a factor is a source of heterogeneity (Higgins et al., 2003). Specifically, if the value of p obtained from the regression analysis for a factor was <0.05, this factor would be a source of heterogeneity, and subgroup analysis of this factor was then performed.

In addition, publication bias was evaluated through visual inspection of funnel plots and formal testing using Egger’s regression test. Significant asymmetry (Egger’s regression test p < 0.10) prompted further sensitivity analysis using the Trim and Fill method (Duval and Tweedie, 2000). Sensitivity analysis was performed to evaluate the robustness of the pooled results by systematically excluding one study at a time to assess the influence of individual trials on the overall effect size (Higgins and Thomas, 2019).

## 3 Results

### 3.1 Search results

**FIGURE 1:**
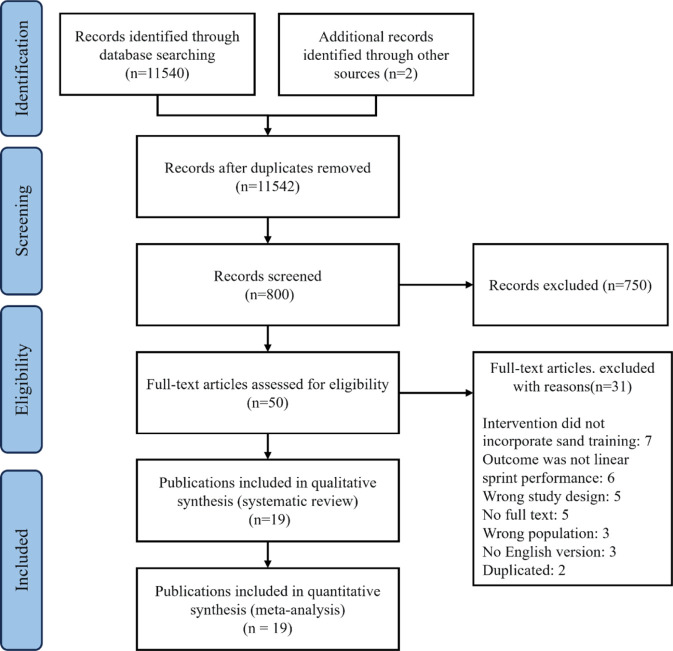
Study flowchart.

The comprehensive search across five electronic databases and supplementary sources identified a total of 11,542 records. Following the removal of 10,742 duplicates, 800 records were screened based on titles and abstracts. This process resulted in the exclusion of 750 studies. The remaining 50 articles were sought for full-text assessment, of which 31 were excluded for the following reasons: absence of SBT intervention (n = 7), lack of linear sprint outcomes (n = 6), inappropriate study design (n = 5), unavailable full text (n = 5), ineligible population (n = 3), non-English language (n = 3), and duplicate publication (n = 2). Ultimately, 19 studies (Impellizzeri et al., 2008; Arazi et al., 2014; Binnie et al., 2014b; Binnie et al., 2013a; de Villarreal et al., 2024; Fernandez-Fernandez et al., 2023; Kumar, 2016; Mehrez et al., 2022; Mina et al., 2021; Mirzaei et al., 2014; Narvariya et al., 2024; Özen et al., 2020; Pereira et al., 2023a; Pereira et al., 2023b; Ramirez-Campillo et al., 2020; Singh et al., 2022; Vuong et al., 2023; Zhang et al., 2024; Pinnington and Dawson, 2001) met the inclusion criteria and were included in this systematic review and meta-analysis (Figure 1).

### 3.2 Risk of bias assessment

**FIGURE 2:**
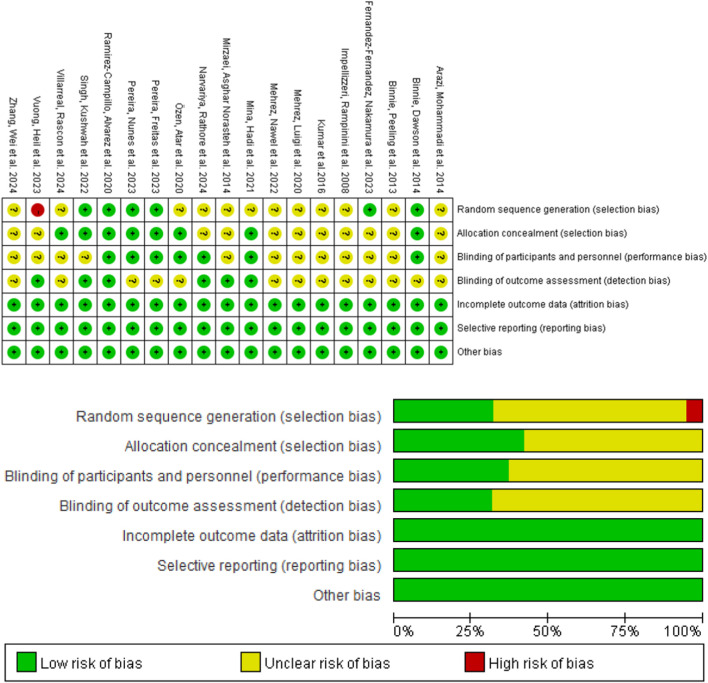
Risk of bias in the included studies.

Risk of bias assessment for the included studies is presented in Figure 2. Due to issues in the randomization process and blinding, most studies were assessed as having some concerns. One study (Vuong et al., 2023) was evaluated as having a high risk of bias, one study (Ramirez-Campillo et al., 2020) was assessed as having a low risk of bias, and the remaining 17 studies (Impellizzeri et al., 2008; Arazi et al., 2014; Binnie et al., 2014b; Binnie et al., 2013a; de Villarreal et al., 2024; Fernandez-Fernandez et al., 2023; Kumar, 2016; Mehrez et al., 2022; Mina et al., 2021; Mirzaei et al., 2014; Narvariya et al., 2024; Özen et al., 2020; Pereira et al., 2023a; Pereira et al., 2023b; Singh et al., 2022; Zhang et al., 2024; Pinnington and Dawson, 2001) were classified as having a moderate risk of bias.

### 3.3 Characteristics of the studies included

The characteristics of the 19 studies included in this systematic review and meta-analysis are summarized in Supplementary S3. These studies investigated the effects of SBT interventions on sprint performance compared to non-sand training or control conditions.

#### 3.3.1 Participants

A total of 433 participants were included across the 19 studies, comprising 355 males, 78 females, and 2 studies (Impellizzeri et al., 2008; Pinnington and Dawson, 2001) that did not disclose gender data. Among these, 221 participants underwent SBT interventions, while 212 served as controls. Participant ages ranged from 16.2 ± 0.4 years (Fernandez-Fernandez et al., 2023) to 25 ± 4 years (Impellizzeri et al., 2008). Most studies included athletes with competitive backgrounds and foundational athletic abilities, including both elite and well-trained athletes (e.g., soccer, volleyball, basketball players). Sample sizes ranged from 11 participants (Pinnington and Dawson, 2001) to 50 participants (Singh et al., 2022), with a median sample size of 22 participants.

#### 3.3.2 Training cycles

The studies were conducted during various training cycles.

Pre-season: 8 studies (Impellizzeri et al., 2008; Binnie et al., 2014b; Binnie et al., 2013a; de Villarreal et al., 2024; Fernandez-Fernandez et al., 2023; Mina et al., 2021; Pereira et al., 2023a; Vuong et al., 2023) implemented sand-based interventions during the pre-season, aiming to prepare athletes for competition.
In-season: 3 studies (Mehrez et al., 2022; Pereira et al., 2023b; Pinnington and Dawson, 2001) integrated SBT into athletes’ regular competition season, focusing on maintaining or enhancing physical performance.
Off-season: 1 study (Özen et al., 2020) conducted interventions during the off-season period, targeting physical conditioning and recovery.
Unspecified: The remaining studies (Arazi et al., 2014; Kumar, 2016; Mirzaei et al., 2014; Narvariya et al., 2024; Ramirez-Campillo et al., 2020; Singh et al., 2022; Zhang et al., 2024) did not specify the training cycle.

#### 3.3.3 Intervention characteristics

The SBT protocols varied in design but predominantly featured plyometric exercises, sprint drills, or combined modalities. Intervention durations ranged from 4 weeks (Narvariya et al., 2024) to 9 weeks (Singh et al., 2022), with a median of 7 weeks. Training frequency ranged from 2 sessions per week (Arazi et al., 2014; Vuong et al., 2023) to 4 sessions per week (de Villarreal et al., 2024) sessions per week. Regarding surface characteristics, the majority of studies standardized sand depth at approximately 20 cm, with one exception (Vuong et al., 2023) utilizing a deeper layer (40∼45 cm).

#### 3.3.4 Comparator groups

The control groups included either non-sand training (e.g., grass, tartan, wooden parquet) or no intervention. Non-sand training programs were matched to sand-based interventions in terms of training frequency, intensity, and duration. Studies with no-intervention control groups, such as Mirzaei et al. (2014), reported only baseline and post-intervention measurements without additional training.

#### 3.3.5 Outcome measures

All included studies assessed linear sprint performance as the primary outcome. Sprint testing distances ranged from 5 m to 60 m. Measurement tools included photocell timing gates, electronic timing systems, and stopwatches, with photocell gates being the most prevalent method.

#### 3.3.6 Sports and athletic levels

Participants across the studies were involved in various sports, including soccer (n = 8), basketball (n = 3), volleyball (n = 2), handball (n = 2), and others (e.g., hockey, tennis). Most studies (n = 16) included well-trained or elite-level athletes, while a few studies (n = 3) involved physically active individuals with a training background (Arazi et al., 2014; Mirzaei et al., 2014; Özen et al., 2020).

#### 3.3.7 Training outcomes

Most studies reported significant improvements in sprint performance within SBT groups compared to baseline. However, between-group analyses yielded mixed findings. Performance enhancements were observed across the full spectrum of sprint distances (5∼60 m).

### 3.4 Results of the meta-analysis

#### 3.4.1 Within-group comparison

**FIGURE 3:**
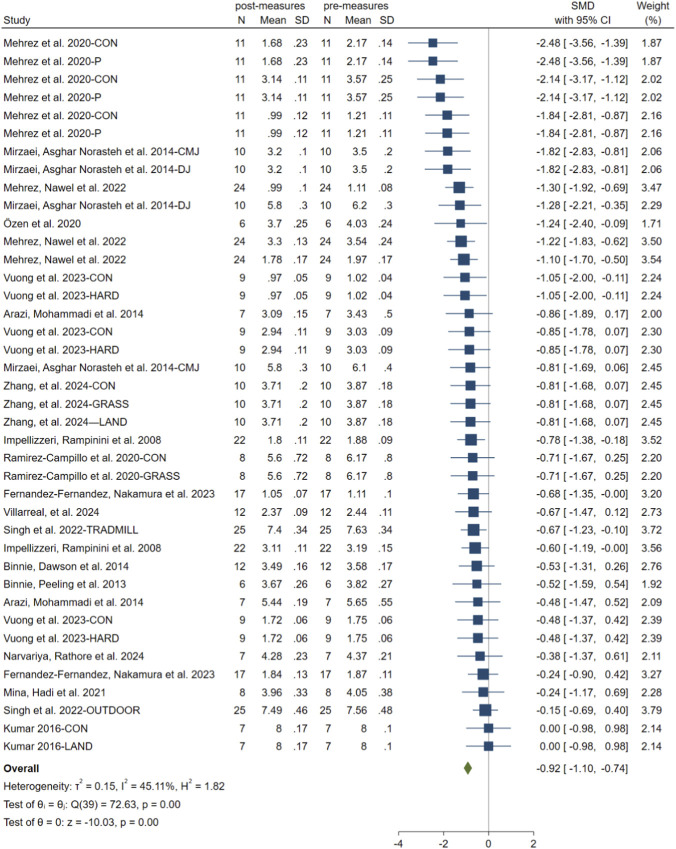
Forest plot comparing pre-measures and postmeasures of linear sprint performance.

The meta-analysis of within-group pre- and post-intervention comparisons showed significant improvements in sprint performance following SBT. Given the substantial heterogeneity observed (I^2^ = 45.11%, p = 0.001), a random-effects model was applied. Across the 19 included studies, the pooled SMD was −0.92 ([95% CI: −1.10 to −0.74], p < 0.001). This indicates a large effect size favoring SBT interventions (Figure 3). The funnel plot and Egger’s test (t = 0.42; p = 0.58) suggested no evidence of publication bias (Figure 5A).

#### 3.4.2 Between-group comparison

**FIGURE 4:**
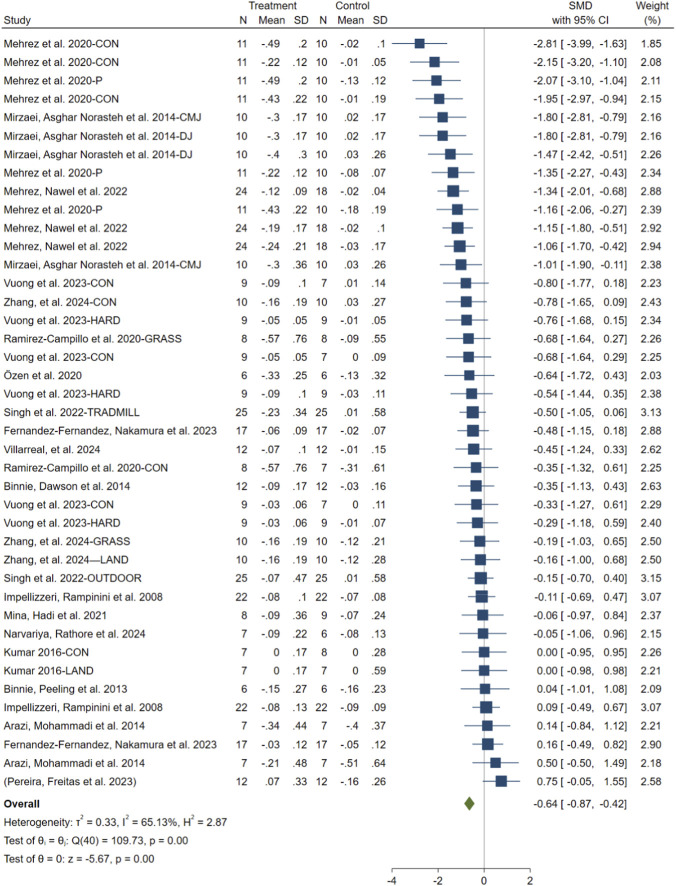
Forest plot comparing prechanges and postchanges among experimental, control, and other conditions.

**FIGURE 5:**
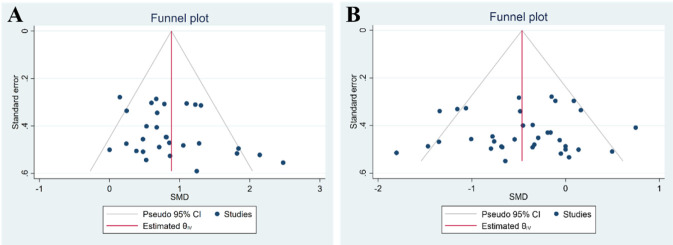
Funnel plots. **(A)** Comparing pre-measures and post-measures of linear sprint performance. **(B)** Comparing prechanges and postchanges among experimental, control, and other conditions.

When comparing SBT groups to control groups (non-sand training or no intervention), significant differences in sprint performance were observed. Given the moderate heterogeneity observed (I^2^ = 65.13%, p = 0.001), a random-effects model was used for the meta-analysis. The pooled SMD for the post-intervention comparisons was −0.64 ([95% CI: −0.87 to −0.42], p < 0.001), indicating a moderate effect size favoring SBT (Figure 4). The funnel plot and Egger’s test (t = 0.35, p = 0.73) suggested no evidence of publication bias (Figure 5B).

#### 3.4.3 Meta-regression and subgroup analysis

Given the substantial heterogeneity observed in the study results (I^2^ = 65.13%), a meta-regression analysis was initially performed to identify potential sources of this heterogeneity. The analysis revealed that several factors significantly contributed to the heterogeneity, including control group type, training frequency, and training cycle (all p < 0.05). In contrast, gender, sprint distance, and training duration did not have a significant impact. Based on the outcomes of the meta-regression, subsequent subgroup analyses were conducted, with the detailed results presented in Table 2.

**TABLE 2:** Subgroup analysis results.

Variables	No. of experimental groups	SMD (95%CI)	*P-* value	Test of heterogeneity		
				I^2^ (%)	χ^2^	P-value
Control group type						
Alternative ground-based training (excluding sand)	26	−0.26 (-0.46, −0.06)	0.01	35.04	1.54	0.03
No-intervention controls	17	−1.13 (-1.45, −0.82)	0.001	52.90	2.12	0.01
Training frequency						
Low frequency (<3 session per week)	20	−0.36 (-0.62, −0.09)	0.01	55.74	2.26	0.001
High frequency (≥3 sessions per week)	23	−0.87 (-1.19, −0.55)	0.001	63.45	2.74	0.001
Training cycles						
Pre-season	14	−0.27 (-0.48, −0.05)	0.01	0.00	1.00	0.86
In-season	12	−1.12 (-1.71, −0.53)	0.001	82.48	5.71	0.001
Off-season	1	−0.64 (-1.72, 0.43)	—	—	—	—

##### 3.4.3.1 Control group type

For control group type, SBT demonstrated a significant and larger effect when compared to no-intervention controls (SMD = −1.13, 95% CI: −1.45 to −0.82, p = 0.001), with moderate heterogeneity (I^2^ = 52.90%, p = 0.01). When compared to alternative ground-based training (excluding sand), SBT also showed a statistically significant but smaller effect size (SMD = −0.26, 95% CI: −0.46 to −0.06, p = 0.01), accompanied by lower heterogeneity (I^2^ = 35.04%, p = 0.03).

##### 3.4.3.2 Training frequency

For training frequency, high-frequency training (≥3 sessions per week) showed a significant and larger effect size (SMD = −0.87, 95% CI: −1.19 to −0.55, p = 0.001), with substantial heterogeneity (I^2^ = 63.45%, p = 0.001). In contrast, low-frequency training (1∼2 sessions per week) also demonstrated a statistically significant effect, though smaller (SMD = −0.36, 95% CI: −0.62 to −0.09, p = 0.01), and had slightly lower heterogeneity (I^2^ = 55.74%, p = 0.001).

##### 3.4.3.3 Training cycle

For training cycle, in-season interventions yielded the largest effect size (SMD = −1.12, 95% CI: −1.71 to −0.53, p = 0.001), though heterogeneity was high (I^2^ = 82.48%, p = 0.001). Pre-season interventions showed a smaller and statistically significant effect size (SMD = −0.27, 95% CI: −0.48 to −0.05, p = 0.01), with negligible heterogeneity (I^2^ = 0.00%, p = 0.86). Off-season interventions demonstrated a moderate effect size (SMD = −0.64, 95% CI: −1.72 to 0.43), but the result did not reach statistical significance.

### 3.5 Sensitivity analysis

Sensitivity analysis demonstrated the robustness of the findings. The pooled results for within-group comparison and between-group comparison remained consistent in both direction and magnitude, with no single study significantly influencing the overall effect sizes (Supplementary Figure 6).

## 4 Discussion

This systematic review and meta-analysis included 19 studies, supporting the effectiveness of SBT in improving sprint performance. Significant improvements were observed in both within-group and between-group analyses, and SBT demonstrated superior efficacy compared to non-intervention control groups and other surface-based training methods. Subgroup analysis identified several key factors influencing the effectiveness of SBT interventions, including training frequency and training cycle. High-frequency training (≥3 times per week) yielded the most significant improvements. Notably, in-season interventions showed the largest and most significant effect sizes, highlighting their particular effectiveness in maintaining or enhancing performance during competitive periods.

Sprint performance is a critical determinant of overall athletic ability, particularly in key skills such as acceleration, sprinting, and shooting (Mero et al., 1992). Identifying strategies that can significantly enhance sprint performance is of great practical importance in sports contexts. Our findings indicate that SBT significantly improved sprint performance. This result aligns with previous research (Pereira et al., 2023a), which demonstrated that after 6 weeks of SBT, linear sprinting performance improved significantly in young team sport athletes. The observed improvement in sprint performance may be attributed to increases in muscle strength and power (Markovic and Mikulic, 2010), suggesting that these physiological adaptations play a key role in enhancing sprinting ability. Regarding differences between surfaces, sand led to higher increases in the first phase of the linear sprint (i.e., 5 m), when the athletes need to effectively accelerate their bodies forward with a higher contribution of concentric strength (Mero et al., 1992). There is evidence that sand is more effective for improving short sprint actions, in which the contribution of muscle contractile capacities seems to be more pronounced (Behm et al., 2017). On the other hand, the improvement in sprints over 5 m can be explained by the fact that while performing explosive actions on firm surfaces may enhance the muscles’ ability to store and utilize elastic energy through SSC (Nicol et al., 2006), SBT increases lower-limb muscle activation levels (Binnie et al., 2014a), leading to improvements in longer sprint distances as well. Additionally, SBT involves numerous acceleration, sprint, and rapid stretch-shortening cycle exercises. The observed improvements following SBT may be attributable to neural adaptations, including enhanced inter-limb coordination and increased stride frequency which lead to significant gains in both eccentric and concentric lower limb muscle strength—an essential prerequisite for improving sprint performance (Negra et al., 2017). Training on sand surfaces requires athletes to exert higher forces during sprints, demanding a more challenging concentric push-off phase to generate high levels of muscle power (Hammami et al., 2020). Given this increased mechanical workload, SBT effectively functions as a supplementary form of resistance training. This potential “strength stimulus” helps explain the significant gains observed in team sport athletes, who may not engage in the highly specialized, velocity-specific resistance protocols typical of elite track sprinters. While world-class sprinters might avoid the low stiffness of sand to preserve maximal velocity mechanics, for team sport athletes, the sand provides a unique, additive strength overload that complements their regular technical training (Markovic and Mikulic, 2010). These adaptations ultimately contribute to a comprehensive improvement in sprint performance. Therefore, SBT provides strong support for significantly enhancing athletes’ sprinting abilities through multiple physiological adaptation mechanisms.

Our findings indicate that SBT significantly improves sprint performance, though the magnitude of the benefit varies depending on the comparator. Specifically, SBT demonstrated a large effect size when compared to no-intervention controls (SMD = −1.13), highlighting the absolute efficacy of the training stimulus. Crucially for competitive contexts, SBT maintained a statistically significant, albeit smaller, advantage (SMD = −0.26) even when compared to alternative training surfaces (e.g., grass or firm ground). This distinction is pivotal: whereas the comparison against inactive controls demonstrates the absolute efficacy of the intervention, the comparison against active controls isolates the specific performance advantage attributable to the sand surface itself. These results are consistent with previous research (Zhang et al., 2024), which showed that in sprint interval training, the sand group (with a 9.6% improvement) outperformed the grass group (7.6%) and the firm ground group (5.2%). The mechanisms driving this “sand-specific” advantage likely stem from distinct physiological and biomechanical differences (Impellizzeri et al., 2008). Training on sand results in higher activation of muscle fibers due to its shock-absorbing and frictional qualities. The instability of the sand surface requires more effort from the muscles to stabilize and push off, leading to increased muscle and tendon work during short sprints. This, in turn, enhances the contractile properties of muscle fibers, improving the sprint performance (Pinnington and Dawson, 2001). The inherent instability of sand may also alter the force vectors exerted during running, potentially leading to adaptations that improve vertical force generation (Pereira et al., 2023b). This adaptation can be attributed to the increased stabilization and force generation needed to navigate the unstable surface (Mirzaei et al., 2014), ultimately resulting in improvements in Sprint Performance. In contrast, training on grass may better align with the principle of training specificity, leading to considerable adaptive changes. While our overall analysis favors sand, it is crucial to acknowledge that recent high-quality trials, such as Pereira et al. (2023b), have found sand and grass surfaces to be equally effective in promoting positive adaptations in linear sprint speed among elite young soccer players. Although the mechanisms behind this result have yet to be fully elucidated, this suggests that for certain populations or specific training protocols, the biomechanical and neuromuscular benefits provided by sand’s instability may be less pronounced compared to the specificity benefits of grass, especially for grass-based sports like soccer and rugby. Thus, while sand and grass both generally provide greater benefits than firm ground, the choice between them may depend on specific training goals and contexts.

High-frequency training (≥3 sessions per week) demonstrated significant training effects in this study, with the underlying mechanisms likely involving multiple factors. First, high-frequency training increases total training volume, providing a stronger cumulative training stimulus that enhances neuromuscular adaptation, including improved motor unit recruitment efficiency, neuromuscular coordination, and muscle endurance (Binnie et al., 2014a; Impellizzeri et al., 2008). Additionally, the high resistance and cushioning properties of SBT further enhance concentric force output during high-frequency training, particularly evident in the sprint start phase. In contrast, low-frequency training (1∼2 sessions per week) also improves sprint performance, but the insufficient training stimulus and longer intervals may limit the maximal gains in muscle strength and neuromuscular adaptation. High-frequency training, by appropriately balancing load and recovery time, mitigates the risk of overtraining while ensuring the accumulation of training effects. These findings provide important insights for the practical application of SBT, indicating that high-frequency sand training can significantly improve sprint performance by enhancing training stimuli and optimizing the recovery balance.

Through subgroup analysis, this study found that SBT during the competitive season had a significant effect, with a SMD of −1.11 (95% CI: −1.71 to −0.51), significantly outperforming interventions conducted in the pre-season and off-season. This result underscores the unique value of SBT as a key method for optimizing athletic performance during the competitive season. The high resistance and low rigidity properties of sand make it particularly effective in absorbing shock during high-intensity training, thereby reducing the impact forces experienced by joints and muscles. This not only mitigates the risk of injuries and soreness but also helps prevent performance decrements associated with fatigue accumulation. Furthermore, the high shock-absorptive qualities of sand can reduce muscle damage and soreness, contributing to better overall performance capacity (Impellizzeri et al., 2008). Further research supports the use of SBT, indicating that it can provide a higher training stimulus without causing additional adverse effects on athletic performance the following day (24 h post-exercise). Specifically, studies have shown that sand surfaces, compared to firm ground, allow for greater relative training intensity while not negatively impacting subsequent performance (Binnie et al., 2013b). These findings highlight the potential of SBT to enhance training efficacy without compromising recovery or performance capacity in the short term. This characteristic makes SBT not only a valuable tool for enhancing athletic performance but also a viable option for fatigue management and injury prevention during the competitive season, particularly in long, high-intensity periods. The unique properties of sand provide an effective training stimulus while simultaneously supporting recovery and rehabilitation, making it an ideal surface for managing the physical demands of the season. In contrast, the effects of SBT during the pre-season and off-season were relatively weaker. This may be attributed to the differing training objectives and characteristics during these phases. In the pre-season, the focus is typically on improving the athlete’s overall physical capabilities, requiring high-intensity and more sport-specific training methods. As a result, the effects of SBT may appear limited in this context. During the off-season, the primary goal is often recovery and adjustment, and the intensity and stimulus provided by SBT may not be sufficient to significantly enhance athletic performance, leading to its more restricted effects.

Several limitations should be noted. First, significant heterogeneity exists among the included studies due to variations in training parameters (e.g., intensity, frequency, sand depth), which may affect the comparability of results. Second, most studies featured small sample sizes and focused on short-term interventions (4∼9 weeks). Consequently, evidence regarding effects across full competitive seasons or multiple training cycles remains unclear, constraining our understanding of the long-term efficacy of SBT. Third, the proportion of male athletes was considerably higher than that of female athletes (355 men vs. 78 women). This substantial gender imbalance restricts the generalizability of our findings, warranting caution when extrapolating the results to female athletes. Finally, regarding the quality of evidence, the majority of included trials were rated as having a “moderate risk” of bias, primarily stemming from deficiencies in the randomization process and blinding. These methodological flaws potentially introduce selection and performance biases, which may lead to an overestimation of the treatment effect. Consequently, this prevalence of moderate risk, along with potential publication bias, necessitates caution when interpreting the robustness of our findings.

Future research should aim to standardize SBT protocols, include more diverse participant demographics, and investigate long-term effects. Additionally, exploring the biomechanical and neuromuscular mechanisms underlying SBT, comparing it with other training methods, and examining its integration into competition periodization can enhance its effectiveness and applicability for a broader range of athletes.

## 5 Conclusion

Our meta-analysis demonstrates that SBT significantly improves sprint performance. However, these findings should be interpreted with caution given that many included studies featured limited sample sizes and duration, as well as presented a moderate risk of bias. The effects of SBT appear to vary depending on several factors, including training frequency, control group type, and training cycles.

These findings highlight the need for a more nuanced approach to SBT implementation, where different variables such as training protocols and athlete characteristics must be carefully considered to maximize the benefits. In the future, further research is needed to explore SBT protocols in greater depth, in order to identify the most effective training plans for enhancing sprint performance.

## Data Availability

The original contributions presented in the study are included in the article/Supplementary Material, further inquiries can be directed to the corresponding author.
