# Supplementary figures and images for: Effect of sand-based training on sprint performance: a systematic review and meta-analysis
Source: Front Physiol. 2026 Feb 16;17:1665495. doi: 10.3389/fphys.2026.1665495 (PMC12950568; doi:10.3389/fphys.2026.1665495)

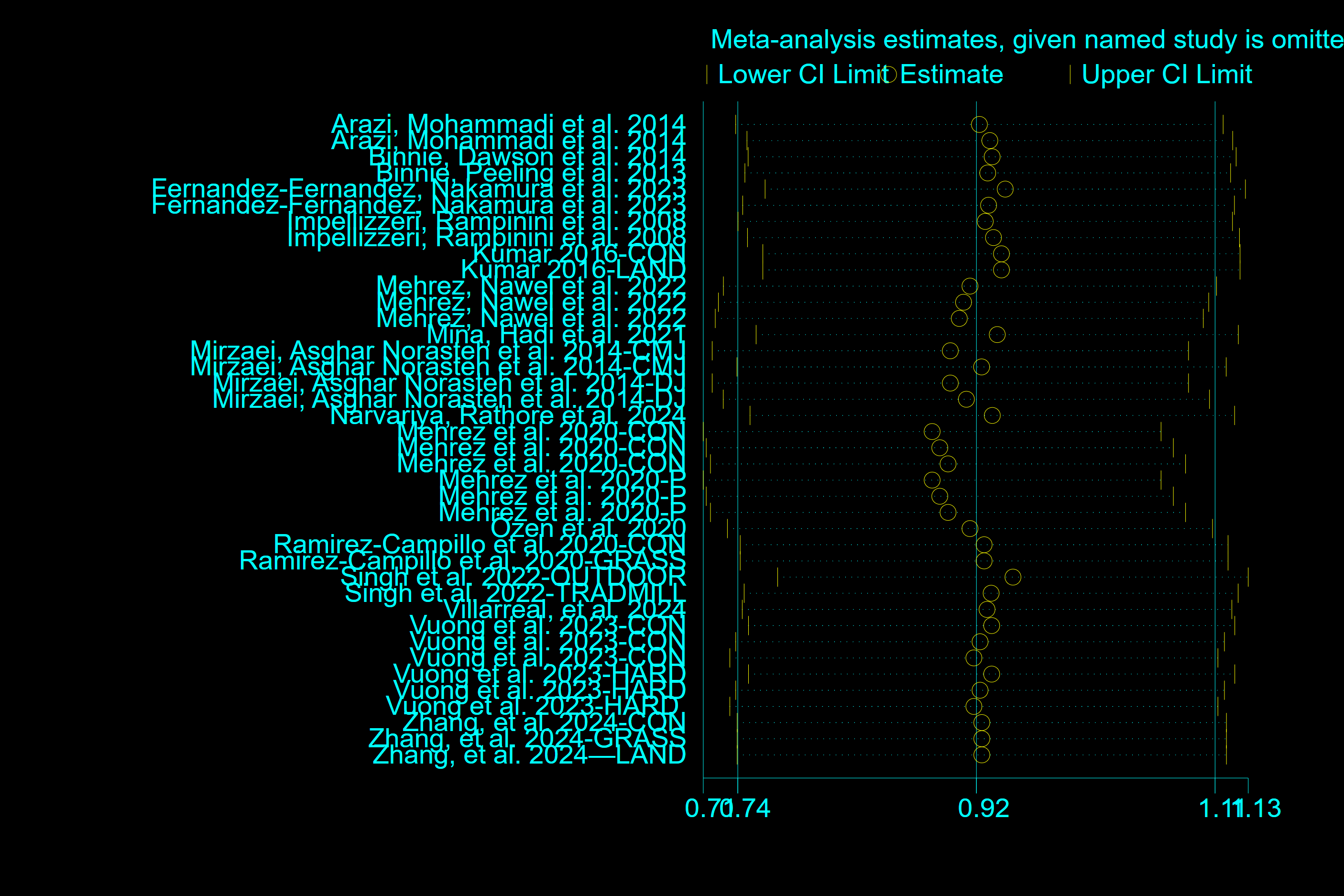

Supplement: Supplementary file 1 [file Image4.tif]

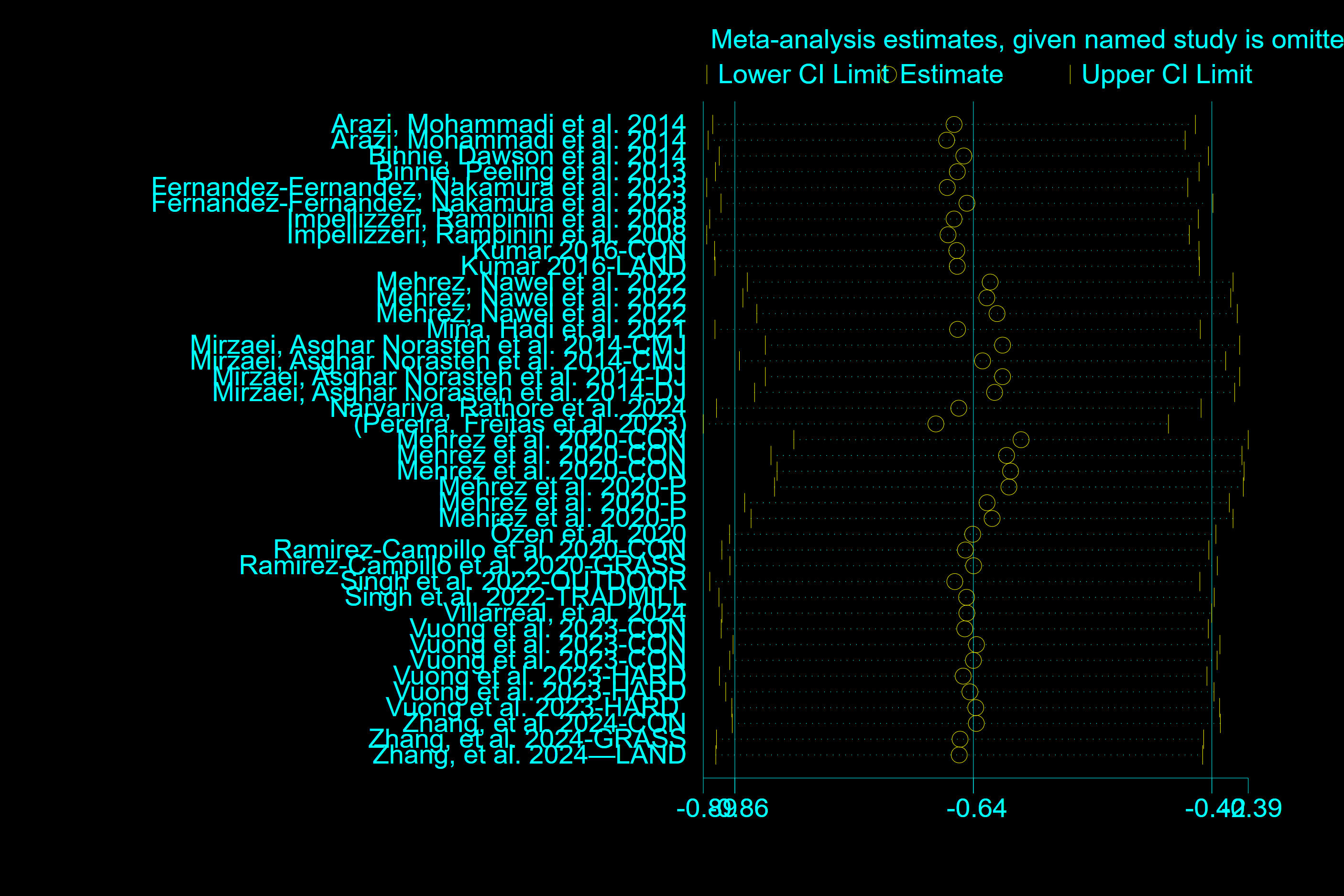

Supplement: Supplementary file 2 [file Image2.TIF]

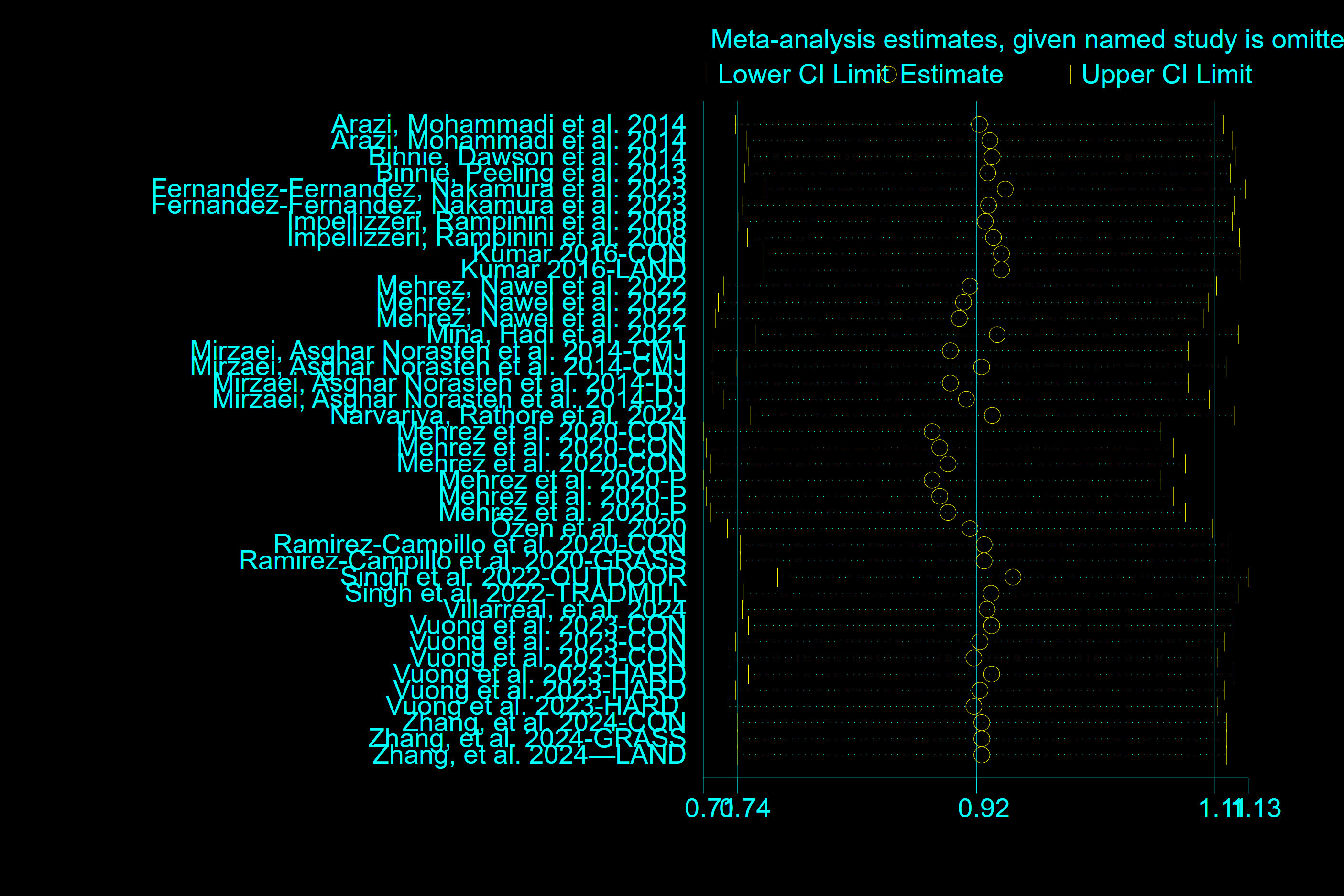

Supplement: Supplementary file 3 [file Image1.TIF]

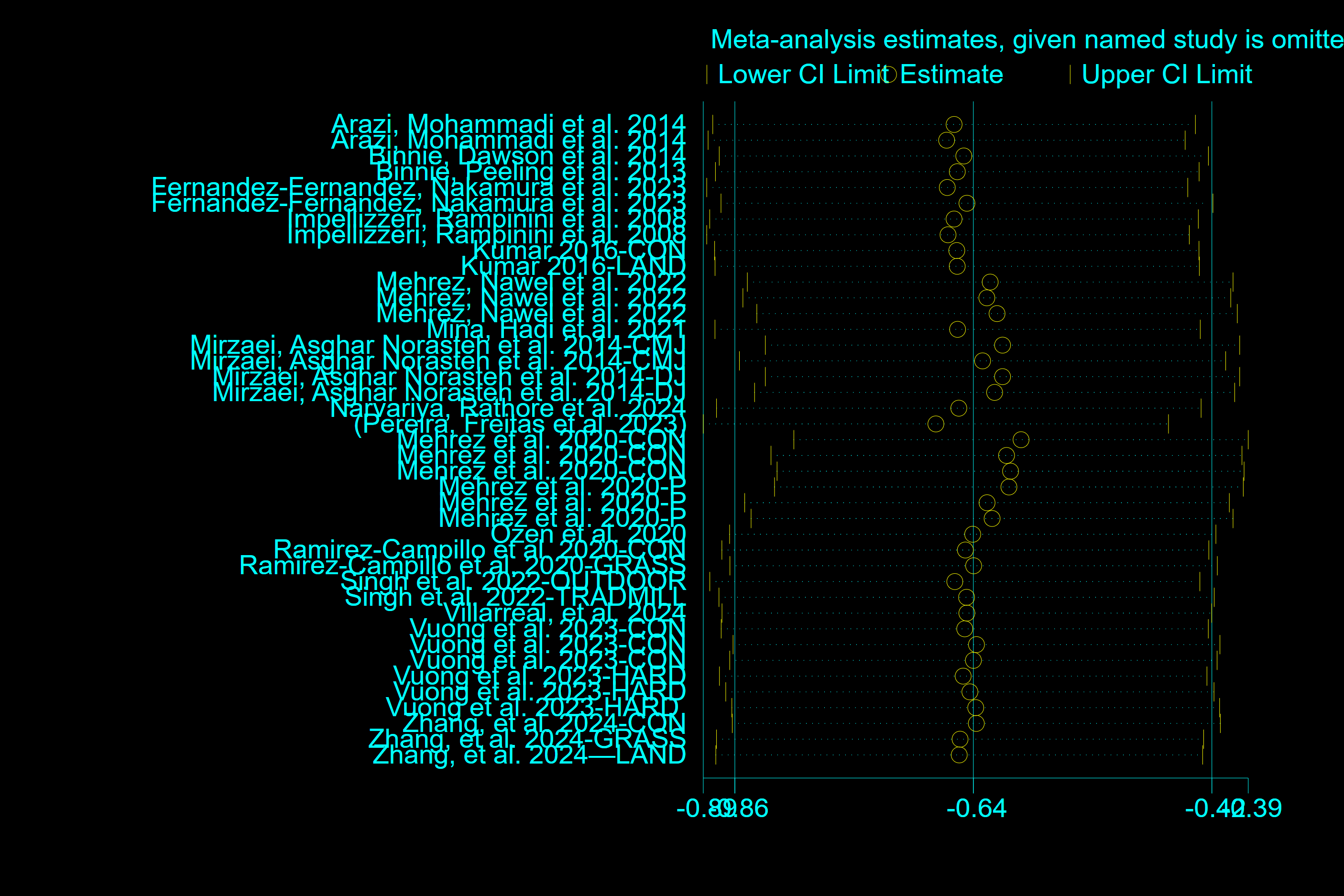

Supplement: Supplementary file 7 [file Image5.tif]
