# Supplementary material for: Effect of sand-based training on sprint performance: a systematic review and meta-analysis
Source: Front Physiol. 2026 Feb 16;17:1665495. doi: 10.3389/fphys.2026.1665495 (PMC12950568; doi:10.3389/fphys.2026.1665495)
Supplement: Supplementary file 4 [file DataSheet1.pdf]

File. Search strategy.

**PubMed/MEDLINE**	(“sand training” [All Fields] OR “sand-based training” [All Fields] OR “beach training” [All Fields] OR “soft surface training” [All Fields] OR “unstable surface training” [All Fields] OR “sand running” [All Fields] OR “sand sprinting” [All Fields] OR “sand volleyball court” [All Fields] OR “sand exercise” [All Fields] OR “Resistance Training/methods”[Mesh] OR “Plyometric Exercise/methods”[Mesh]) AND ((“sprint performance”[All Fields] OR “sprint time”[All Fields] OR “sprint speed”[All Fields] OR “acceleration”[All Fields] OR “linear sprint”[All Fields] OR “short sprint”[All Fields] OR (“10m”[Title/Abstract] OR “20m”[Title/Abstract] OR “30m” [Title/Abstract] OR “40m”[Title/Abstract]) OR (“agility”[All Fields]AND (“test” [All Fields] OR “performance” [All Fields])) NOT (“long-distance” [All Fields] OR “endurance” [All Fields] OR “marathon” [All Fields])) AND ((“systematic review” [Publication Type] OR “meta-analysis” [Publication Type] OR “systematic literature review” [All Fields] OR “evidence synthesis” [All Fields]) OR (“review” [Publication Type] AND (“systematic” [Title/Abstract] OR “meta-analysis” [Title/Abstract])))
**Web of Science (Core Collection)**	(TS=((athletes OR players OR participants OR subjects OR “physically active” OR men OR women)))AND (TS=((train* OR intervention*) AND(“sand training” OR “sand-based training” OR “sand based training” OR “beach training” OR “sand surface training” OR “training on sand” OR “sand running” OR “running on sand” OR “sprinting on sand” OR “sand exercise” OR “sand workouts” OR “soft surface training”)))AND (TS=((“sprint performance” OR “sprint time” OR “sprint speed” OR “sprint velocity” OR “sprint test” OR “sprint ability” OR “running speed” OR “short sprint” OR “short-distance sprint”)))NOT (SILOID==(“PPRN”))
**Scopus**	((TITLE-ABS-KEY (“sand training”) OR TITLE-ABS-KEY (“sand-based training”) OR TITLE-ABS-KEY (“beach training”) OR TITLE-ABS-KEY (“sand surface”) OR TITLE-ABS-KEY (“training on sand”) OR TITLE-ABS-KEY (“sand exercise”) OR TITLE-ABS-KEY (“sand running”) OR TITLE-ABS-KEY (“sand sprinting”))AND ((TITLE-ABS-KEY (“sprint performance”) OR TITLE-ABS-KEY (“sprint time”) OR TITLE-ABS-KEY (“sprint speed”) OR TITLE-ABS-KEY (“sprint test”) OR TITLE-ABS-KEY (“sprint ability”) OR TITLE-ABS-KEY (“acceleration”) OR TITLE-ABS-

	KEY (“linear sprint”) OR TITLE-ABS-KEY (“short sprint”) OR TITLE-ABS-KEY (“sprint training”)))
**Cochrane library**	#1 (“sprint*” OR “short-distance run*” OR “athlet*” OR “runner*” OR “sprinter*” OR “physically active” OR “team sport*” OR “field sport*”)<br>#2 (“sand train*” OR “sand-based train*” OR “sand surface train*” OR “beach train*” OR “soft surface train*” OR “unstable surface train*” OR “sand run*” OR “sand sprint*”)<br>#3 (“sprint performance” OR “sprint time” OR “sprint speed” OR “sprint velocity” OR “acceleration” OR “sprint test” OR “short sprint*” OR “20m sprint” OR “30m sprint” OR “40m sprint” OR “60m sprint” OR “100m sprint”)<br>#4 #1 AND #2 AND #3
**SPORTDiscus**	S1(“sand-based training” OR “sand training” OR “sand surface training” OR “sandy terrain training” OR “beach training” OR “unstable surface training” OR “sand running” OR “sand drills”)<br>S2 (“sprint performance” OR “sprint ability” OR “sprint test” OR “sprint time” OR “sprint speed” OR “sprint velocity” OR “acceleration” OR “running speed” OR “10m sprint” OR “20m sprint” OR “30m sprint”)<br>S3 S1 AND S2
**Google Scholar**	(“sand training” OR “sand-based training” OR “beach training” OR “unstable surface training”)<br>AND (“sprint performance” OR “sprint speed” OR “sprint time” OR “20m sprint” OR “30m sprint” OR “acceleration” OR “running velocity”)
