# Supplementary material for: Effect of sand-based training on sprint performance: a systematic review and meta-analysis
Source: Front Physiol. 2026 Feb 16;17:1665495. doi: 10.3389/fphys.2026.1665495 (PMC12950568; doi:10.3389/fphys.2026.1665495)
Supplement: Supplementary file 5 [file Table3.docx]

# Table 3. Characteristics of sand-based training and participants

| **Study** | **Age^a^ (year)** | **Fitness^b^** | **Sports** | **No. of participants** | | **Quantity** | | **Training Cycles** | **Eercise program** | | **Duration (week)** | **Frequency**  **(session/week)** | **Sprint** | **Sprint measure** |
| --- | --- | --- | --- | --- | --- | --- | --- | --- | --- | --- | --- | --- | --- | --- |
|  |  |  |  | **M** | **F** | **EXP** | **CON** |  | **EXP** | **CON** |  |  |  |  |
| Arazi *et al*. (38) | 20.7±0.5 | Physically active with training background | —— | 14 | 0 | 7 | 7 | —— | Sand surface (20-cm dry sand) | Hard court surface | 6 | 2 | 20 m sprint↓⮆  40 m sprint↓⮆ | Electronic timing gates |
| Binnie *et al*. (39) | 20~24 | Well-trained team-sport athletes | Field Hockey and Netball programs | 2 | 10 | 6 | 6 | pre-season | Sand surface | Grass surface | 8 | 3 | 20 m sprint ↓⮆ | Electronic timing gates |
| Binnie *et al*. (40) | 19~26 | Well-trained female team sport athletes | Netball and Field Hockey | 0 | 24 | 12 | 12 | pre-season | Sand surface | Grass surface | 8 | 3 | 20 m sprint↓⮇ | Electronic timing gates (SMARTSPEED) |
| Impellizzeri *et al*. (19) | 25±4 | Well-trained female team sport athletes | soccer | —— | —— | 22 | 22 | pre-season | Sand surface (20-cm dry sand) | Grass surface | 4 | 3 | 10 m sprint↓⮆  20 m sprint↓⮆ | Electronic timing gates |
| Mehrez *et al*. ((25)-P) | 16.5±0.4 | Al-saniyah club players | handball | —— | —— | 11 | 10 | in-season | performing plyometrics on dry sand | Gymnasium floor | 7 | 3 | 5 m sprint↓⮇  10 m sprint↓⮇  20 m sprint↓⮇ | Electronic timing gates |
| Mehrez *et al*. ((25)-CON) | 16.5±0.4 | Elite-level championship | handball | —— | —— | 11 | 10 | in-season | performing plyometrics on dry sand | The control group did not train. | 7 | 3 | 5 m sprint↓⮇  10 m sprint↓⮇  20 m sprint↓⮇ | Electronic timing gates |
| Mehrez *et al*. (41) | 16.3±0.4 | Elite-level championship | handball | 40 | 0 | 24 | 18 | in-season | Sand surface | Hard court surface | 7 | 3 | 5 m sprint↓⮇  10 m sprint↓⮇  20 m sprint↓⮇ | Electronic timing gates |
| Mina *et al*. (42) | 23.5±2.8 | National U17 team | volleyball | 17 | 0 | 8 | 9 | pre-season | Sand surface | Hard court surface | 8 | 3 | 20 m sprint↓⮆ | Single-beam timing gates |
| Özen *et al*. (43) | 17.58±0.5 | Physically active with training background | volleyball | 12 | 0 | 6 | 6 | off-season | Sand surface (20-cm dry sand) | 10-cm-thick wooden surface | 6 | 3 | 30 m sprint↓⮇ | Electronic timing gates |
| Pereira *et al*. (44) | 18.3±0.5 | Well-trained young male players | Soccer | 24 | 0 | 12 | 12 | pre-season | Sand surface | Grass surface | 6 | 2 | 20 m sprint→⮆ | Electronic timing gates |
| Ramirez-Campillo *et al*. ((45)-grass) | 12±2 | Elite young athletes played | soccer | 15 | 0 | 8 | 8 | —— | Sand surface | Grass surface | 8 | 3 | 30 m sprint↓⮇ | Single-beam timing gates |
| Ramirez-Campillo *et al*. ((45) -CON) | 12±2 | Regional-level soccer team. | soccer | 15 | 0 | 8 | 7 | —— | Sand surface | The control group did not train | 8 | 3 | 30 m sprint↓⮇ | Single-beam timing gates |
| Singh *et al*. ((46)-outdoor) | 20.1±1.7 | Collegiate male athletes | —— | 50 | 0 | 25 | 25 | —— | Sand surface | Treadmill surface | 9 | 1~2 | 50 m sprint↓⮇ | Stopwatch |
| Singh *et al*.((46)-treadmill) | 20.1±1.7 | Collegiate male athletes | —— | 50 | 0 | 25 | 25 | —— | Sand surface | Treadmill surface | 9 | 1~2 | 50 m sprint↓⮇ | Stopwatch |
| Villarreal, *et al*. (47) | 23.0±4.9 | Collegiate male athletes | beach handball players | 24 | 0 | 12 | 12 | pre-season | Sand surface | Hard court surface | 6 | 4 | 15 m sprint↓⮆ | Electronic timing gates |
| Vuong *et al*.((48)-CON) | 24.2±4.6 | National-level basketball player | basketball | 16 | 0 | 9 | 7 | pre-season | Sand surface (40~45-cm dry sand) | The control group did not train. | 7 | 2 | 5 m sprint↓⮆  10 m sprint↓⮆  20 m sprint↓⮇ | Electronic timing gates |
| Vuong *et al*. ((48)-hard) | 24.2±4.6 | National-level basketball player | basketball | 18 | 0 | 9 | 9 | pre-season | Sand surface (40~45-cm dry sand) | Hard court surface | 7 | 2 | 5 m sprint↓⮆  10 m sprint↓⮆  20 m sprint↓⮇ | Electronic timing gates |
| Zhang, *et al*. ((49)-LAND) | 21.6±2.3 | National-level basketball player | soccer | 0 | 20 | 10 | 10 | —— | Sand surface (20-cm dry sand) | Grass surface | 7 | 3 | 20 m sprint↓⮆ | Single-beam timing gates |
| Zhang, *et al*.((49)-GRASS) | 21.6±2.3 | Collegiate female athletes | soccer | 0 | 20 | 10 | 10 | —— | Sand surface (20-cm dry sand) | Grass surface | 7 | 3 | 20 m sprint↓⮆ | Single-beam timing gates |
| Zhang, *et al*. ((49)-CON) | 21.6±2.3 | Collegiate female athletes | soccer | 0 | 20 | 10 | 10 | —— | Sand surface (20-cm dry sand) | Hard court surface | 7 | 3 | 20 m sprint↓⮆ | Single-beam timing gates |
| Kumar *et al* ((50) -land) | 18~24 | Collegiate male athletes | hockey players | 14 | 0 | 7 | 7 | —— | Sand surface | Hard court surface | 8 | 3 | 50 m sprint↓⮆ | —— |
| Kumar *et al* ((50) -con) | 18~24 | Collegiate male athletes | hockey players | 15 | 0 | 7 | 8 | —— | Sand surface | The control group did not train | 8 | 3 | 50 m sprint↓⮇ | —— |
| Mirzaei *et al*. ((51)-DJ) | 20.4±1.1 | Physically active with training background | —— | 30 | 0 | 10 | 10 | —— | Sand surface | The control group did not train | 6 | 2 | 20 m sprint↓⮆  40 m sprint↓⮆ | Stopwatch |
| Mirzaei *et al*.((51)-CMJ) | 20.4±1.1 | Physically active with training background | —— | 30 | 0 | 10 | 10 | —— | Sand surface | The control group did not train | 6 | 2 | 20 m sprint↓⮆  40 m sprint↓⮆ | Stopwatch |
| Narvariya *et al*.(52) | 20.3±1.5 | Collegiate male athletes | soccer | 13 | 0 | 7 | 6 | —— | Sand surface | Grass surface | 4 | 2 | 30 m sprint→⮆ | Electronic timing gates |
| Pereira *et al*. (53) | 18.5±0.6 | Elite young soccer players | soccer | 15 | 0 | 7 | 8 | in-season | Sand surface | Grass surface | 8 | 1~2 | 10 m sprint→⮆  17 m sprint↓⮆ | Contact platform |
| Fernandez-Fernandez *et al*. (54) | 16.2±0.4 | Highly trained tennis player | tennis | 34 | 0 | 17 | 17 | pre-season | Sand surface | Hard court surface | 6 | 2 | 5 m sprint↓⮇  10 m sprint↓⮆ | Single-beam timing gates |

↓, EXP significantly(p<0.05) reduced the outcome compared to PRE; ↑, EXP significantly(p<0.05) improved the outcome compared to PRE; →, no significant difference (p> 0.05) between POST and PRE; ⮇, EXP significantly(p<0.05) reduced the outcome compared to CON; ⮅, EXP significantly(p<0.05) improved the outcome compared to CON; ⮆, no significant difference (p> 0.05) between EXP and CON; ——, no mentioned.

a Mean values for experimental/control groups (mean ± SD are reported for those studies where authors reported combined data for experimental and control groups).

b Fitness was classified here as it was in the recent review by Ramirez-Campillo (64): (1) NR; (2) high encompasses professional/elite athletes with regular enrollment in national and/or international competitions, or highly trained participants with 10 training hours/week or 6 training sessions/week and a regularly scheduled official or friendly competition; (3) moderate encompasses non-elite/professional athletes with a regular attendance in regional and/or national competitions, between 5.0~9.9 training hours/week or 3~5 training sessions/week and a regularly scheduled official or friendly competition; and (4) normal encompasses recreational athletes with <5 training hours/week with sporadic or no participation in competition.
